# Potential of Pullulan-Based Polymeric Nanoparticles for Improving Drug Physicochemical Properties and Effectiveness

**DOI:** 10.3390/polym16152151

**Published:** 2024-07-29

**Authors:** Nurain Thomas, Lisa Efriani Puluhulawa, Faradila Ratu Cindana Mo’o, Agus Rusdin, Amirah Mohd Gazzali, Arif Budiman

**Affiliations:** 1 Department of Pharmacy, Faculty of Sport and Health, Universitas Negeri Gorontalo, Jl. Jenderal Sudirman No. 6, Gorontalo 96128, Indonesia; nurain.thomas@ung.ac.id (N.T.); lisapuluhulawa@ung.ac.id (L.E.P.); faradilaratu@ung.ac.id (F.R.C.M.); 2 Department of Pharmaceutics and Pharmaceutical Technology, Faculty of Pharmacy, Universitas Padjadjaran, Jl. Raya Bandung-Sumedang Km. 21, Bandung 45363, Indonesia; agusrusdin@gmail.com; 3 Department Pharmaceutical Technology, School of Pharmaceutical Sciences, Universiti Sains Malaysia, P.Penang, Penang 11800, Malaysia; amirahmg@usm.my

**Keywords:** pullulan, nanoparticles, biopolymer, drug delivery, drug targeting

## Abstract

Pullulan, a natural polysaccharide with unique biocompatibility and biodegradability, has gained prominence in nanomedicine. Its application in nanoparticle drug delivery systems showcases its potential for precision medicine. Aim of Study: This scientific review aims to comprehensively discuss and summarize recent advancements in pullulan-based polymeric nanoparticles, focusing on their formulation, characterization, evaluation, and efficacy. Methodology: A search on Scopus, PubMed, and Google Scholar, using “Pullulan and Nanoparticle” as keywords, identified relevant articles in recent years. Results: The literature search highlighted a diverse range of studies on the pullulan-based polymeric nanoparticles, including the success of high-selectivity hybrid pullulan-based nanoparticles for efficient boron delivery in colon cancer as the active targeting nanoparticle, the specific and high-efficiency release profile of the development of hyalgan-coated pullulan-based nanoparticles, and the design of multifunctional microneedle patches that incorporated pullulan–collagen-based nanoparticle-loaded antimicrobials to accelerate wound healing. These studies collectively underscore the versatility and transformative potential of pullulan-based polymeric nanoparticles in addressing biomedical challenges. Conclusion: Pullulan-based polymeric nanoparticles are promising candidates for innovative drug delivery systems, with the potential to overcome the limitations associated with traditional delivery methods.

## 1. Introduction

The dynamic landscape of pharmaceutical advancement has lead to the continuous evolution of drug delivery systems as a way to increase treatment effectiveness and patient compliance [1,2,3,4]. Currently, many targeted and controlled drug delivery methods have been developed, ranging from conventional pharmaceutical dosage forms like solid, liquid, and semisolid [5,6,7,8] to sophisticated nanoparticle carriers [9,10,11]. These advances not only offer opportunities for personalized medicine, but also overcome challenges such as poor bioavailability, off-target effects, and drug resistance [12,13].

In recent years, the field of drug delivery systems has experienced extraordinary progress, including the development of nanoparticle systems. Nanoparticle-based drug delivery systems offer excellent advantages such as passive and active targeted drug delivery and controlled release, which can increase bioavailability [14,15,16]. In this field, polymeric nanoparticles have received great attention due to the flexibility, biocompatibility, and other properties of polymers that are advantages of such systems [17,18]. One type of polymer that is often used in polymer nanoparticle systems is pullulan. Pullulan is a promising polymer for the development of nanoparticle drug delivery systems, considering its potential to improve physicochemical properties and good drug performance [19].

Pullulan, a natural polysaccharide derived from fermented starch, has attracted much attention because it is a versatile polymer for nanoparticle-based drug delivery systems. Pullulan has unique properties, including its biocompatibility and biodegradability, and can provide modifiable drug release kinetics [19,20,21]. It is these advantages that make it an attractive candidate for improving the effectiveness and safety of therapeutic agents. Pullulan-based nanoparticles have been widely developed, and the results of these developments demonstrate that pullulan offers nanoparticles with controlled drug release, targeted delivery, and increased drug stability, thereby overcoming key challenges in pharmaceutical formulation [22,23,24].

Although several published studies investigated the use of pullulan polymers in nanoparticle drug delivery systems, a comprehensive review summarizing their successful applications and their potential benefits is still lacking. Although previous review articles focused on specific aspects such as anticancer drug delivery or transmucosal pulmonary delivery [22,25], there is an important gap in the literature regarding the comprehensive analysis of pullulan as a primary nanoparticle drug delivery system. 

Therefore, this review article aims to fill this gap by providing a comprehensive overview of pullulan-based nanoparticle systems and their implications for drug delivery. The main aim of this review article is to critically evaluate the current state-of-the-art pullulan nanoparticle-based drug delivery systems, synthesize the existing literature, and highlight key findings. By examining the successes and challenges associated with pullulan as a polymer matrix for drug delivery, this study seeks to provide valuable insights for researchers, physicians, and pharmaceutical professionals involved in the development of innovative therapeutic strategies.

## 2. Pullulan: A Polymer Powerhouse

### 2.1. Overview of Pullulan’s Properties and Characteristics

Due to its attractive properties and characteristics, pullulan has potential in a variety of applications. Chemically, pullulan consists of repeating units of maltotriose, which are linked through α-1,6 glycosidic bonds, thus making it a linear homopolysaccharide [26]. High molecular weight (typically between 200,000 and 2,000,000 Da) is seen in pullulan, and this results in superior film forming, moisture retention, and viscosity [27]. Pullulan is transparent, water-soluble, and virtually tasteless; therefore, it has broad applications in different areas including food, pharmaceutical, and cosmetic products [28]. As a film former, pullulan is excellent as an edible coating to prolong the shelf life of fruits and vegetables, and in the pharmaceutical industry, pullulan is used to encapsulate bioactive compounds [29]. Perhaps the most attractive property of pullulan is its remarkable film strength, oxygen barrier properties, and biodegradability, besides being an environmentally friendly alternative. It is also a non-toxic and non-allergenic material, and, as it is compatible with most materials, it has a wide range of potential applications [30]. For example, pullulan is used to form complexes with a range of molecules, including proteins and polyphenols [31]. Its potential as a drug delivery system is particularly exciting due to its valuable properties as a biomaterial.

The characterization of pullulan nanoparticles includes morphology, particle size, zeta potential, and advanced characterization analyses, such as amorphous and crystalline analysis using XRD and melting point analysis using DSC. Particle size is crucial in designing and evaluating nanoparticle formulations, influencing their behavior and efficacy in biomedical applications. Analyzing data from studies on pullulan-based nanoparticles reveals their potential for drug delivery, wound healing, antimicrobial therapy, and cancer treatment [32]. The morphology of nanoparticles is crucial for their performance and functionality in various applications. Studies have shown varied morphologies of pullulan-based nanoparticles, influencing their properties and uses. Nanoparticles maintained their shape post-incorporation into tablets, indicating a stable formulation. The dynamic change in morphology is useful for controlled drug release. Overall, tailoring nanoparticle morphology enhances properties like drug release, cellular uptake, stability, and antimicrobial activity, which are crucial for biomedical and environmental applications [33,34,35].

The combined analysis of XRD, DSC, and FTIR results across the articles confirms the successful formation of pullulan-based polymeric nanoparticles. XRD shows crystallinity and structural integrity, with sharp peaks indicating crystalline nanoparticles. DSC analysis reveals thermal characteristics, confirming nanoparticle stability, such as consistent melting points and distinct thermal peaks. FTIR confirms the chemical composition and functionalization of nanoparticles. Overall, XRD, DSC, and FTIR analyses collectively validate the formation and potential applications of pullulan-based nanoparticles in drug delivery, wound healing, and antimicrobial therapy [36].

### 2.2. Biocompatibility and Safety of Pullulan in Medical Applications

Pullulan is a naturally occurring polysaccharide derived from microbial fermentation which has gained significant attention due to exhibiting biocompatibility [37], biodegradability [38], and low immunogenicity [39]; its possession of such ideal characteristics make it an excellent candidate for utilization in drug delivery applications. Its ability to form stable complexes with drugs through various techniques such as encapsulation, conjugation, or blending has been extensively explored [40,41,42]. 

A comprehensive exploration of pullulan’s safety and biocompatibility in medical applications is crucial to its success for a myriad of biomedical applications. Pullulan is a naturally occurring polysaccharide from the starch fermented by Aureobasidium pullulans and has attracted attention owing to its biocompatibility, non-toxicity, and biodegradability [43,44]. The majority of medical uses, including medication delivery [45,46], wound dressings [47], and scaffolds for tissue engineering [48], require an understanding of how it interacts with biological systems. Its hydrophilicity permits high drug loading and controlled release, and its physical properties can be tailored to design scaffolds for tissue regeneration [49]. Furthermore, pullulan is broken down into non-toxic by-products by enzymatic reactions and is harmlessly cleared from the body. However, challenges such as optimizing its mechanical properties for specific applications and enhancing its stability in physiological environments remain actively investigated areas. Addressing these concerns through interdisciplinary research will further enhance the understanding and utilization of pullulan in medical applications.

The safety profile of pullulan in medical applications is supported by extensive research and its history of use in the food and pharmaceutical industries. Numerous key studies, both preclinical and clinical, have demonstrated pullulan’s safety in various formulations, including drug delivery systems, wound dressings, and tissue engineering scaffolds. Pullulan has been approved by regulatory bodies like the FDA for use in food products, which underscores its safety. This approval facilitates its acceptance in medical and pharmaceutical applications [50,51].

### 2.3. Previous Applications of Pullulan in Drug Delivery Systems

Previous applications and successes of pullulan in drug delivery systems have showcased its versatility and efficacy in various pharmaceutical applications [52]. Pullulan-based drug delivery systems have demonstrated enhanced solubility, controlled release profiles, improved stability, and the targeted delivery of therapeutics [53]. Additionally, pullulan can be modified to tailor its properties for specific applications, including surface modification for targeted delivery [54] and the incorporation of stimuli-responsive elements for triggered release [55]. Furthermore, studies have demonstrated pullulan-based formulations’ potential for administering an array of therapeutics, having effectively conveyed small molecules [56], peptides [57], proteins [58], and nucleic acids [59]. 

Pullulan’s biocompatibility and safety make it ideal for various medical applications. Pullulan nanoparticles and microneedles enhance drug bioavailability and efficacy while reducing systemic side effects. Pullulan-based films and hydrogels provide a moist environment and infection protection, promoting healing without irritation. Additionally, pullulan scaffolds support cell growth and tissue regeneration, making them suitable for reconstructive surgery and regenerative medicine. Functionalized pullulan coatings prevent infections on medical devices without compromising safety [45,60].

## 3. Nanoparticle Drug Delivery Systems

### 3.1. The Advantages of Nanoparticles for Drug Delivery

The ability of nanoparticles to deliver drugs is recognized as the cutting edge in pharmaceutical studies as this capacity holds promise for the advancement of medicine [61,62]. Because of their small size—typically between 1 and 100 nanometers—nanoparticles have specific qualities that could help to improve drug delivery [63,64,65]. Due to their disproportionately large surface area relative to size, these structures allow for the meticulous oversight of the kinetics of pharmaceutical distribution, while also enabling the proficient containment and dispersion of remedial agents, the process by which a nanoparticle enters a cell, attaches itself to the cell’s outer membrane, and then becomes internalized by the cell. Naturally, for the nanoparticle to be internalized—that is, to avoid desorbing—it must stay at the membrane for a sufficient amount of time. According to the research, the nanoparticles adsorb to the cell quickly—within 10 to 20 min [66]. Moreover, nanoparticles can be engineered to display particular physicochemical characteristics, such as surface charge, shape, and surface functionalization, which minimizes systemic toxicity and enables targeted delivery to diseased cells and tissues [67,68,69]. Moreover, their bioavailability and therapeutic efficiency are improved by their capacity to elude immune detection by surface modifications using ligands or polymers, such as polyethylene glycol (PEG), and their ability to cross biological barriers like the blood–brain barrier (BBB) [70,71,72]. Furthermore, because of their versatile construction, nanoparticles can be used to incorporate imaging agents for theranostic applications or to co-deliver several medications [73,74,75]. Pharmacokinetics and patient compliance are further optimized by their capacity for regulated medication release profiles and sustained release. Nanotechnology has enabled the synthesis of stimuli-responsive nanoparticles with the potential to precisely release encapsulated drugs upon the detection of specific environmental changes such as pH, temperature, or enzyme levels, thus enhancing the effectiveness of therapeutic regimens [76]. In addition, by enabling intracellular drug delivery and improving the stability of drugs in the laboratory, nanoparticles can solve the problems of traditional drug formulations. Increasing the stability of drugs that are labile due to nanoparticles can encapsulate labile drugs; protecting them from environmental factors can create a barrier against water, reducing hydrolysis; and encapsulation can protect and shield drugs from oxygen and light exposure [77,78]. While the benefits are indeed impressive, ongoing studies continue exploring matters such as long-term safety profiles, reproducibility at greater scales, and the ability to expand the approach [79]. Although the numerous advantages that nanoparticles provide for drug delivery highlight their potential to revolutionize the therapeutics field by providing innovative approaches to treating a variety of illnesses, there are still some difficult obstacles to overcome before this potential can be realized.

### 3.2. Comparison between Conventionall Drug Delivery Methods and Nanoparticle

There are various conventional ways to transport drugs to the target cell, but all these methods have certain limitations [80]. One of the main challenges in the treatment of many diseases is moving the therapeutic chemical to the intended location. Drugs used conventionally typically have poor biodistribution, little selectivity, and limited efficacy. The management of drug delivery can help to overcome these constraints and disadvantages.

By delivering the medication to the site of action through controlled drug delivery systems (DDS), the impact of the drug on important tissues and unwanted side effects can be reduced. Moreover, DDS improves drug concentration in target tissues and shields the medication against quick degradation or evacuation, necessitating lower dosages. This cutting-edge type of therapy is particularly crucial when a drug’s therapeutic effects do not match its dose or concentration [81].

Drugs can be targeted to specific cells by attaching them to specially made carriers. Recent advancements in nanotechnology have demonstrated the enormous potential of nanoparticles, which are structures smaller than 100 nm in at least one dimension, as medication carriers. The nanostructures are a desirable material for biomedical applications because of their small sizes and distinctive physicochemical and biological features (such as an increased reactive area and the capacity to pass cell and tissue barriers) [81].

Nanoparticle-based delivery systems (NDS) provide several benefits over conventional drug delivery techniques. The targeting efficiency is enhanced through nanoparticles’ unique capacity to reach specific cells, tissues, or organs within the body, thereby potentially decreasing unintended reactions elsewhere and optimizing therapeutic benefits [82]. Among others, the advantages of NDS include the following: (1) Decreased systemic toxicity: Systemic toxicity and side effects may be reduced by limiting the exposure time of the healthy tissues towards high drug concentrations [83]. (2) Enhanced stability: medications with weak physicochemical qualities can benefit especially from the protection that nanoparticles provide against degradation in biological fluids. This improves the stability and bioavailability of the medications [84,85]. (3) Extended circulation time: By altering the surface of nanoparticles, their bloodstream circulation time can be extended, resulting in increased therapeutic benefits and prolonged drug release [86]. (4) Overcoming biological barriers: Drug distribution to previously inaccessible regions and certain diseased tissues is made possible by nanoparticles’ ability to circumvent biological barriers like the blood–brain barrier and intestinal epithelium [87]. (5) Tailored formulations: The formulation design flexibility provided by nanoparticle-based systems enables the customization of drug release kinetics, targeting ligands, and other features to match the needs of patients [88,89].

### 3.3. Role of Pullulan in Enhancing the Efficacy of Nanoparticle-Based Drug Delivery

Pullulan, a naturally occurring polysaccharide generated from starch, has drawn significant focus because of its potential to augment the efficacy of drug delivery. Due to the unique physicochemical properties it possesses, including its mucoadhesiveness, biocompatibility, and biodegradability, it is highly compatible with living tissues, making it an ideal candidate for various pharmaceutical applications [28,29]. Pullulan is a versatile structure that can be used to functionalize chemical reactions and create the possibility of hydrogen bonding or electrostatic hydrophobic interactions with other small-molecule composites. It is possible to tune the hydrophilic–hydrophobic equilibrium and physical interactions in pullulan derivatives to provide drug-loading controlled systems for drug delivery to cancer cell and tissue receptors [22].

Pullulan can be added to nanoparticle formulations to increase stability, extend the in vivo circulation time, and improve drug-loading efficiency [90,91]. Additionally, pullulan-coated nanoparticles have shown better tissue penetration and increased cellular absorption. These results are explained by pullulan’s mucoadhesive quality, which encourages attachment to mucosal surfaces and enables effective drug delivery to target tissues [92,93]. Additionally, therapeutic drugs can also be released under regulated conditions using pullulan-based nanoparticles. This results in prolonged drug release profiles that maximize therapeutic efficacy while reducing systemic toxicity [94]. Furthermore, pullulan has an innate capacity to influence cellular absorption pathways and interact with biological membranes to increase the effectiveness of delivery [95]. All things considered, the use of pullulan in nanoparticle formulations offers a viable approach to overcoming the challenges associated with traditional drug delivery techniques and raising the effectiveness of therapeutic interventions in a variety of biological applications.

## 4. Recent Developments in Pullulan-Polymer Nanoparticles 

Currently, the development of drug delivery systems has been widely studied, one of which is the development of polymeric nanoparticles. Various types of polymers have been developed, one of which is pullulan. Here are some lists of pullulan nanoparticle developments that have been carried out.

**Table 1:** Recent developments in pullulan-based polymeric nanoparticles.

Nanoparticle	Preparation Method	Primary Aims	Particle Size	Zeta Potential/PDI	Result	Ref.
Polymeric	Bottom-up method	Develop a hybrid nanoparticle for efficient boron delivery in cancer therapy	-	-	- HBNGs demonstrated superior anti-cancer effects on Colon26 cells compared to the L-BPA/fructose complex. - High tumor selectivity observed with HBNGs due to enhanced permeation and retention effects.	[94]
Polymeric	Dialysis method	Create biodegradable nano-formulation for enhanced delivery and efficacy of ferulic acid	425 ± 5.2 nm	-	- Nano-formulation showed high encapsulation efficiency and substantial drug release improvement. - Negligible toxicity and significant cytotoxicity against gastrointestinal cancer cell lines were observed.	[96]
Polymeric	Bottom-up method	Design a smart microneedle patch for differential drug release to aid wound healing	258.0 ± 10.86 nm	45.1 ± 3.9 mV/0.19 ± 0.06	- Smart microneedle patch demonstrated rapid wound healing with collagen deposition and accelerated cell proliferation. - Potential for multifunctional wound healing with sustained bactericidal action.	[97]
Polymeric	Bottom-up method	Develop redox-sensitive prodrug nanoparticles for targeted delivery of paclitaxel in cancer therapy	134–163 nm	-	- Redox-sensitive prodrug nanoparticles exhibited improved tumor-suppressing properties with reduced systemic toxicity. - Enhanced targeting efficiency and augmented anticancer potential demonstrated.	[98]
Polymeric	Bottom-up method	Prepare pullulan nanoparticles loaded with methotrexate and camptothecin for synergistic tumor-targeted therapy	185.7 ± 16.7 nm	-	- Dual drug-loaded nanoparticles exhibited enhanced killing ability against HeLa cells. - Potential carrier for tumor-targeted synergistic therapy with sustained drug release profile.	[99]
Polymeric-Folate Conjugated	Dialysis method without a surfactant	Construct a non-spherical drug delivery system based on folate-conjugated pullulan acetate for placental targeting	-	-	- Folate-conjugated nanoparticles showed placental targeting and translocation with good biocompatibility and cellular uptake. - Potential for drug use during pregnancy and placental-mediated therapy.	[100]
Polymeric-Albumin	Self-assembly	Fabricate EGCG-loaded nanoparticles for enhanced stability and antioxidant activity	-	-	- EGCG-loaded nanoparticles exhibited high encapsulation efficiency and antioxidant activity. - Continuous release profile and stable carrier for enhancing EGCG stability and antioxidant activity.	[101]
Polymeric-Trastuzumab Functionalized	Bottom-up method	Develop trastuzumab functionalized nanoparticles for active targeting of HER-2 positive breast cancer cells	66.7 ± 2.0 nm	PDI: 0.218 ± 0.012	- Trastuzumab-functionalized nanoparticles showed significant cell uptake and cytotoxic effect against HER-2 positive breast cancer cells. - Potential candidate for active targeting HER-2 positive breast cancer treatment.	[86]
Polymeric-Themoresponsive	Bottom-up method	Synthesize thermoresponsive hydrogel loaded with pullulan nanoparticles for sustained drug release	-	-	- Thermoresponsive hydrogel loaded with nanoparticles showed an enhanced sustained release drug profile. - Superior pharmacokinetic parameters observed compared to non-targeted nanoparticles.	[102]
Polymeric-Complex Inclusion	The emulsion solvent evaporation method	Design a liver-specific drug delivery system using glycyrrhetinic acid-grafted pullulan nanoparticles	200 nm	-	- Liver-specific drug delivery system demonstrated significant cellular uptake and anticancer therapeutic effect. - Potential for liver-specific drug delivery and improved anticancer efficacy.	[103]
Polymeric-Amino Acid	Dialysis method	Develop drug-loaded nanoparticles based on cholesterol-modified carboxylated pullulan for controlled drug release	118.7 nm	-	- Hydrophobically modified nanoparticles exhibited a slow-release effect and significant cytotoxicity against cancer cells. - Strongest cytotoxicity observed with nanoparticles having the highest hydrophobic substitution.	[104]
Polymeric-Cholesterol	Dialysis method	Design cholesterol-modified carboxylated pullulan nanoparticles for controlled release and cytotoxicity evaluation	178.0 nm	-	- Cholesterol-modified nanoparticles showed slow drug release and hydrophobic substitution degree-related cytotoxicity against hepatoma cells. - Strongest inhibitory effect observed with nanoparticles having the lowest hydrophobic substitution.	[105]
Metal Nanoparticle	Bottom-up method	Synthesize stable gold nanoparticles embedded in a tablet for glucose monitoring	-	-	- AuNPs-pTab exhibited stability for >6 months and showed better peroxidase-mimic enzymatic behavior than AuNPs-pSol. - Promising for glucose assay and disease diagnosis with high recovery rates.	[95]
Metal Nanoparticle	Green synthesis	Synthesize silver nanoparticles using green method and evaluate their antimicrobial and catalytic properties	20 nm	-	- Green-synthesized AgNps showed good antibacterial activity and biocompatibility. - Efficient photocatalysis in degrading water-soluble pollutants observed. - No cytotoxic effect on human dermal fibroblast cells.	[33]
Metal Nanoparticle	Green synthesis	Synthesize pullulan decorated with Ag and Au nanoparticles and evaluate their antimicrobial and QS inhibition effects	-	-	- Pullulan-decorated nanoparticles exhibited enhanced antimicrobial activity and quorum sensing inhibition. - Potential therapeutic approach against bacterial resistance.	[106]

### 4.1. Characterization of Physicochemical Properties 

#### 4.1.1. Particle Size and Zeta Potential

Particle size is a pivotal factor in the design and evaluation of nanoparticle formulations as it profoundly influences their behavior and efficacy in various biomedical applications. Analyzing the particle size data from a selection of studies focusing on pullulan-based nanoparticles provides invaluable insights into their potential for drug delivery, wound healing, antimicrobial therapy, and cancer treatment. In the study by Varadhan and Jayaraman (2024), hyalgan-coated polymeric pullulan nanoparticles were synthesized with a particle size of 425 ± 5.2 nm (Table 1). Despite being relatively large, this size falls within the nanoscale range, suggesting potential benefits in terms of the enhanced permeability and retention (EPR) effect in tumor tissues. Similarly, Younas et al. (2023) reported pullulan-based microneedle patches loaded with moxifloxacin (MOX)-loaded nanoparticles, which exhibited a particle size of 258.0 ± 10.86 nm. The nanoparticles showed effective penetration into skin layers for drug delivery applications [100]. In contrast, Zhao et al. (2023) developed redox-sensitive pullulan/paclitaxel-based prodrug nanoparticles with an average size range of 134–163 nm. These relatively smaller nanoparticles are advantageous for facilitating cellular uptake and improving drug delivery efficiency, particularly in cancer therapy [101]. Constantin et al. (2023) synthesized oxidized pullulan-capped silver nanoparticles with an ultra-small size of approximately 20 nm, which is beneficial for antimicrobial and photocatalytic activities due to the increased surface area-to-volume ratio [102]. Furthermore, Xu et al. (2022) reported trastuzumab functionalized pullulan-doxorubicin nanoparticles with a hydrodynamic diameter of 66.7 ± 2.0 nm. This size is ideal for targeting HER-2 overexpression in breast cancer cells, ensuring efficient cellular uptake and targeted drug delivery [90]. Similarly, pullulan nanoparticles loaded with MTX and HCPT as reported by Wu et al. (2022) had a particle size of 185.7 ± 16.7 nm which is suitable for tumor-targeting with reduced off-target delivery [103]. Moreover, Tian et al. (2022) and Wu et al. (2022) synthesized nanoparticles with sizes ranging from 118.7 nm to 229.4 nm [104] and 178.0 nm to 229.4 nm [106], respectively, which facilitates their cellular uptake and biodistribution. In summary, the reported particle sizes of pullulan-based nanoparticles across these studies demonstrate their versatility and potential for various biomedical applications. Despite variations in size, all nanoparticles fall within the nanoscale range. 

Ghaffarlou et al. (2022) reported that silver (AgNPs) and gold nanoparticles (AuNPs) capped with pullulan (Pull) exhibited zeta potentials of −23.2 ± 4.85 mV and −17.9 ± 6.92 mV, respectively, which are significantly more negative than the pristine Pull’s zeta potential of −2.98 ± 4.31 mV [106]. This indicates that the reduction in metal ions and the oxidation of pullulan’s hydroxyl groups to carboxylate contribute to the increased negative surface charge. In contrast, Wypij et al. (2023) found that silver nanoparticles capped with biomolecules from the F. culmorum strain JTW1 had a zeta potential of −30.1 mV. This more negative value suggests that biomolecular capping provides substantial stability by preventing nanoparticle aggregation [29]. Similarly, Jayeoye et al. (2023) observed that pullulan-capped gold nanoparticles (PUL-AuNPs) had a zeta potential of −15.4 ± 0.8 mV, attributed to the deprotonated carboxyl groups of pullulan, indicating effective stabilization [107]. Roy et al. (2021) measured a zeta potential of −25.2 ± 0.5 mV for nanoparticles in aqueous conditions, indicating moderate stability [108]. On the extreme end, Salleh et al. (2021) reported a highly negative zeta potential of −72.05 ± 0.93 mV for silver nanoparticles (Ag-NP/PL), suggesting exceptional stability due to significant electrostatic repulsion [109]. These findings emphasize that more negative zeta potentials generally correlate with increased nanoparticle stability. The degree of negative charge varies depending on the capping agents and surface modifications, with biomolecular and polymeric capping providing effective stabilization by enhancing the negative surface charge and preventing aggregation. This comparative analysis underscores the critical role of zeta potential in determining the colloidal stability and functional performance of nanoparticles in various applications.

#### 4.1.2. Morphology Study

The morphology or shape of nanoparticles plays a crucial role in determining their performance and functionality in various applications. In the studies reviewed, different approaches were employed to synthesize pullulan-based nanoparticles, resulting in varied morphologies that influence their properties and applications. Al-Kassawneh et al. (2022) observed a similar morphology between nanoparticles in a solution and those incorporated into tablets [95]. This maintenance of shape post-incorporation suggests a stable formulation, potentially contributing to the prolonged stability and enhanced performance of the nanoparticles in applications such as drug delivery or sensing. The preservation of morphology is crucial for ensuring the consistent behavior and efficacy of the nanoparticles over time. In contrast, studies by Jiang et al. (2022) and Li et al. (2022) focused on achieving specific morphologies tailored for targeted delivery applications. Jiang et al. (2022) adjusted synthesis parameters to obtain non-spherical nanoparticles, which would likely facilitate enhanced cellular uptake and translocation across biological barriers [100]. The authors suggested that the tailored morphology enhances the efficacy of the nanoparticles in targeted drug delivery, particularly for applications such as placental targeting in pregnancy-related treatments. On the other hand, Zhao et al. (2023) demonstrated the rapid dismantling of self-assembled architecture in response to reducing conditions, leading to a triggered drug release [98]. This dynamic change in morphology from a compact structure to a dispersed form is critical to achieving controlled drug release, enabling the precise delivery of therapeutic agents to the target sites while minimizing off-target effects. Furthermore, Constantin et al. (2023) synthesized pullulan silver nanoparticles with a small size and round shape, enhancing their stability and antimicrobial activity [33]. The well-controlled synthesis process likely contributed to the uniform morphology, optimizing the performance of the nanoparticles in applications such as antibacterial coatings or water treatment. Overall, the morphology of pullulan-based nanoparticles significantly influences their performance in various applications. Tailoring the morphology allows for the customization of properties such as drug release kinetics, cellular uptake, stability, and antimicrobial activity, leading to enhanced efficacy and versatility in biomedical and environmental applications. Understanding and controlling nanoparticle morphology is essential for harnessing the full potential of pullulan-based nanoparticles in diverse fields of research and technology [110].

#### 4.1.3. FTIR Analysis

FTIR analysis serves as a cornerstone in confirming the chemical composition and functionalization of nanoparticles. By identifying characteristic peaks corresponding to pullulan and any modifications or conjugations, FTIR offers definitive proof of successful nanoparticle synthesis. According to Titilope et al. (2023) with the title “Toxic Ag^+^ detection based on Au@ Ag core shell nanostructure formation using Tannic acid assisted synthesis of Pullulan stabilized gold nanoparticles”, FTIR analysis offers significant advantages in the development of pullulan-based polymer nanoparticles. Firstly, FTIR spectroscopy enables the identification of characteristic functional groups of pullulan within the nanoparticle structure. Pullulan, being a polysaccharide, exhibits distinct vibrational bands in the FTIR spectrum such as O-H, C-H, and C-O bonds, among others. This capability allows for the verification of pullulan’s presence as the main component in the nanoparticles. Secondly, FTIR is crucial for monitoring the synthesis process of pullulan nanoparticles. During synthesis, FTIR detects changes in vibrational spectra that indicate the formation of chemical bonds between pullulan and other additives, like tannic acid (TA) and Ag^+^ ions, as studied in the article. This information is essential to ensure proper synthesis yielding nanoparticles with desired compositions. Additionally, FTIR assists in characterizing interactions between pullulan and additives such as TA and Ag^+^. By examining shifts or changes in the FTIR spectra following Ag^+^ addition, insights are gained into how Ag^+^ interacts with pullulan nanoparticles. This helps in designing and understanding reaction mechanisms that determine the properties and performance of the nanoparticles. Furthermore, FTIR serves both qualitative (functional group identification) and quantitative analysis purposes. For instance, in the study, changes in FTIR intensity at 409 nm after Ag^+^ addition were used to measure Ag^+^ concentrations with a low detection limit. Lastly, FTIR provides insights into the structure and surface properties of pullulan nanoparticles, which are crucial for understanding stability, reactivity, and interactions in practical applications, such as Ag^+^ detection in water and other environmental applications. Thus, FTIR spectroscopy stands as a powerful tool in the development of pullulan-based polymer nanoparticles, offering deep insights into chemical composition, additive interactions, and other physicochemical characteristics which are vital for environmental sensing and technological applications. 

Similarly, Swarup et al. (2021) FTIR analysis offers several advantages in the development of pullulan-based nanoparticles. Firstly, FTIR spectroscopy facilitates the identification of characteristic functional groups of pullulan, a polysaccharide composed of glucose monomers linked via alpha(1 → 4) and alpha(1 → 6) bonds. This capability enables the verification of pullulan’s presence within the nanoparticles. Studies such as Li et al. (2022) utilize FTIR to confirm the presence of specific functional groups driving the formation of EGCG-loaded nanoparticles, providing direct evidence of successful nanoparticle system formation [101]. 

#### 4.1.4. X-ray Diffraction Analysis

X-ray Diffraction (XRD) analysis is an essential technique for characterizing the crystallinity and structural properties of materials at the nanoscale. This summary synthesizes findings from several studies that applied XRD to examine silver nanoparticles (AgNPs) and gold nanoparticles (AuNPs) synthesized via various methods and embedded in polymer matrices such as pullulan. 

XRD elucidates the crystallinity and structural integrity of nanoparticles, offering distinct patterns that signify successful synthesis. For instance, studies, such as that from Constantin et al. (2023), demonstrate sharp XRD peaks which are characteristic of crystalline metallic nanoparticles embedded within pullulan matrices, affirming their stable formation. Conversely, in studies focusing on drug-loaded polymeric nanoparticles, like Y. Wu et al. (2022), XRD might reveal broader peaks which are indicative of the amorphous nature of the polymeric matrix, corroborating successful nanoparticle formation [105].

Ghaffarlou et al. (2022) identified six main peaks for AgNPs@Pull at 2θ values of 38.0°, 46.6°, 53.4°, 56.3°, 65.7°, and 79.8°, corresponding to the (111), (200), (210), (211), (220), and (311) planes, respectively. For AuNPs@Pull, four peaks were observed at 38.2°, 44.5°, 64.7°, and 77.7°, corresponding to the (111), (200), (220), and (311) planes [106]. Similarly, Salleh’s (2020) study reported four main peaks for AgNPs/PL at 2θ values of 38.20°, 44.0°, 64.20°, and 77.40°, corresponding to the (111), (200), (220), and (311) planes. These results align with the theoretical standard (JCPDS file no. 01-087-0718) [111].

M. Wypij (2023) used XPS to analyze the surface composition of pullulan films enriched with AgNPs. It found that the addition of AgNPs at various concentrations affected the nitrogen and carbon content but did not detect silver on the surface, suggesting the uneven distribution or degradation of AgNPs during analysis [29]. Jayeoye’s (2023) XPS analysis of TA/PUL-AuNPs showed peaks for Au 4f, C 1s, and O 1s spectra. The presence of both Au0 and Au^+^ indicated some unreduced Au salts. The study also confirmed strong interactions between AuNPs, TA, and PUL, forming a stable nanocomposite network [107].

Salleh (2020) and Salleh (2021) both emphasized the high purity and crystallinity of AgNPs/PL, with XRD patterns showing sharp and well-defined peaks, which are indicative of nanoscale particles and minimal impurities. These studies used XRD to investigate the reaction mechanisms and confirmed that the incorporation of Ag-NPs and the irradiation techniques transformed pullulan from an amorphous to a crystalline state [109,111]. Ghaffarlou’s (2022) study also confirmed the crystalline structure of AgNPs and AuNPs, with well-defined peaks indicating high purity and alignment with theoretical standards [106].

The XRD analyses from these studies consistently demonstrate the formation of well-defined crystalline structures in synthesized AgNPs and AuNPs embedded in pullulan. The observed diffraction patterns align with theoretical standards, confirming the high purity and crystallinity of the nanoparticles. However, surface composition analysis using XPS revealed challenges in the even distribution and detection of nanoparticles on the surface, suggesting areas for further improvement in the synthesis process. 

#### 4.1.5. DSC Analysis

Differential Scanning Calorimetry (DSC) plays a crucial role in characterizing pullulan nanoparticles loaded with bioactive compounds. Firstly, DSC is used to determine the melting point of these nanoparticles, providing insights into their thermal stability and suitability for applications requiring specific temperature ranges, such as in controlled drug delivery systems. Secondly, DSC enables the monitoring of interactions between pullulan as the polymer matrix and additives. Changes in the calorimetric profiles indicate any physical or chemical interactions, which are vital for assessing the stability and functionality of the nanoparticle formulations. Additionally, despite pullulan’s generally amorphous nature, DSC can detect the formation of crystalline phases within the nanoparticles, if present, thereby influencing their structural and physicochemical properties. Lastly, DSC allows for thermodynamic studies such as heat capacity and enthalpy measurements, providing quantitative data on the energy involved in thermal transitions. This information aids in optimizing synthesis processes and ensuring the stability of pullulan nanoparticles, thereby enhancing their efficacy as carriers for bioactive compounds in biomedical applications. Furthermore, DSC analysis unveils crucial thermal characteristics, reinforcing the integrity of the nanoparticle systems. In investigations such as Al-Kassawneh et al. (2022), where gold nanoparticles are integrated into pullulan matrices, DSC could demonstrate consistent melting or phase transition temperatures, affirming the stability of the nanoparticle system [81]. Similarly, Tian et al. (2022) might utilize DSC to reveal distinct endothermic or exothermic peaks corresponding to drug-loaded nanoparticles, providing evidence of successful nanoparticle formation and stability under varying thermal conditions [91].

### 4.2. Stability, Solubility, and Drug Release Behavior

#### 4.2.1. Stability Study

Analyzing the stability studies across the reviewed articles showed several important factors that influence the performance and potential applications of pullulan-based polymeric nanoparticles. Among the studies, Al-Kassawneh et al. (2022) demonstrated that pullulan-gold nanoparticle exhibited superior stability, maintaining stability for over six months [95]. This enhanced stability is attributed to the solid-state matrix provided by the tablet formulation, effectively protecting the nanoparticles from degradation or aggregation. Similarly, Constantin et al. (2023) reported no significant agglomeration of oxidized pullulan-capped silver nanoparticles (OxP-AgNps) over six months, highlighting the efficacy of pullulan as a capping agent in preventing aggregation [33]. 

Furthermore, Y. Wu et al. (2022) observed consistent drug release rates of cholesterol-modified carboxylated pullulan (CHSP) polymer-based nanoparticles over 72 h, indicating robust stability even under weakly acidic conditions [105]. Additionally, Zhao et al. (2023) demonstrated the dynamic structural changes in redox-sensitive pullulan/paclitaxel-based prodrug nanoparticles (PULL-SS-PTX NPs) under reducing conditions while maintaining outstanding colloidal stability under physiological conditions [98]. These findings collectively underscore the reliability and potential applications of pullulan-based polymeric nanoparticles. The prolonged stability observed in these studies suggests their suitability for long-term storage and use, while their ability to maintain structural integrity under physiological conditions highlights their potential for effective and controlled drug delivery. This correlation between stability and performance emphasizes the importance of stable formulations in achieving the desired therapeutic outcomes and ensuring the practical applicability of pullulan-based nanoparticles in biomedical and environmental fields.

#### 4.2.2. Solubility Study and Drug Release

Varadhan and Jayaraman’s study (2024) provides a detailed analysis of the solubility and drug release characteristics of their pullulan-based nanoparticle formulation [96]. They conducted solubility tests to assess the ability of the nanoparticles to dissolve in various media, such as water and physiological solutions. This is crucial as it determines the effectiveness of the nanoparticles in delivering drugs to target tissues and cells. The results of their solubility tests indicate that the pullulan nanoparticles exhibit high solubility in these media, suggesting their suitability for drug delivery applications. Regarding the drug release test, the authors also conducted a series of in vitro assays to evaluate the release kinetics of the encapsulated drug from the nanoparticles. They monitored the release of the drug over time and analyzed the data to determine the rate and extent of drug release. Their findings demonstrated the ability of pullulan nanoparticles in controlled drug release, with most of the drug being released within a specific time frame. This controlled release is advantageous as it allows for precise dosing and prolonged therapeutic effects, thus minimizing the risk of drug toxicity and enhancing patient compliance [96].

Zhao et al. (2023) also reported an investigation on the solubility of pullulan nanoparticles in different media and their ability to dissolve and release the encapsulated drug. The results of their solubility tests indicated a favorable solubility property; in terms of drug release, the nanoparticles are responsive to specific stimuli such as reducing conditions, which allow for targeted and controlled drug delivery [98]. Tian et al. (2022) modified amino–pullulan polymers with polyethylene glycol to form sustained-release nanoparticles, whereby PCCN1 exhibited the strongest cytotoxicity [104]. Y. Wu et al. (2022) also designed cholesterol-modified carboxylated pullulan nanoparticles with varying degrees of substitution, which demonstrates slow drug release profiles that can be correlated with the degree of hydrophobic substitution. On the other hand, a higher cytotoxicity profile against hepatoma cells was observed in the nanoparticles prepared from pullulan with lower hydrophobic substitution [105]. 

Overall, the studies provide a comprehensive analysis of the solubility and drug release characteristics of their pullulan-based nanoparticle formulations. Based on the results obtained, the release and solubility profile of each nanoparticle differs depending on the formulation technique and materials used [112,113]. However, essentially all formulations demonstrated improved solubility profiles and controlled drug release.

### 4.3. Drug Effectiveness 

Pullulan-based polymeric nanoparticles have demonstrated significant potential in enhancing drug effectiveness and performance across various studies. Kawasaki et al. (2023) introduced hybrid nanoparticles comprising carborane-bearing pullulan nanogel and hydrophobized boron oxide nanoparticles (HBNGs), which exhibited improved anti-cancer effects on Colon26 cells compared to a clinically used boron agent. This enhancement was attributed to the increased accumulation and retention of the boron agent within cells, leading to higher tumor selectivity and therapeutic efficacy surpassing conventional agents. This study stands out for its significant advancement in cancer therapeutics, achieving superior anti-cancer effects and tumor regression [94].

Similarly, Al-Kassawneh et al. (2022) synthesized AuNPs-pullulan that shows better peroxidase-mimic enzymatic behavior and stability compared to AuNPs-pSol. It provides an excellent substitute for solution-phase nanoparticles with enhanced catalytic efficiency [95]. Varadhan and Jayaraman (2024), on the other hand, formulated ferulic acid-loaded pullulan nanoparticles that demonstrate improved drug release kinetics, biocompatibility, and cytotoxicity against cancer cell lines [96]. Pullulan nanoparticles have also been incorporated into smart microneedle patches. Younas et al. (2023) developed moxifloxacin-loaded nanoparticles in microneedle patches that facilitated rapid wound healing through the sustained release of the encapsulated antibiotic [97]. Zhao et al. (2023) engineered redox-sensitive pullulan-based prodrug nanoparticles, achieving targeted drug delivery with improved tumor-suppressing properties and reduced systemic toxicity. These studies collectively showcase the versatility and efficacy of pullulan-based nanoparticles in enhancing drug delivery and therapeutic outcomes [98]. 

Regarding the mechanisms of action, the following is an in-depth discussion on how pullulan-polymer nanoparticles work. Pullulan-based polymeric nanoparticles exhibit intricate mechanisms at the molecular level that significantly impact their characteristics and enhance drug effectiveness in various biomedical applications. In the study by Kawasaki et al. (2023), the incorporation of carborane-bearing pullulan nanogel in hybrid nanoparticles played a pivotal role in efficient boron delivery for boron neutron capture therapy (BNCT) [94]. The pullulan nanogel not only served as a carrier for boron agents, but also contributed to the improved accumulation and retention of these agents within cancer cells. This mechanism was attributed to the enhanced permeation and retention effect, resulting in heightened anti-cancer effects by surpassing the limitations of clinically used boron agents. The molecular-level interaction between pullulan and boron within the nanogel matrix played a crucial role in achieving high tumor selectivity.

Similarly, Varadhan and Jayaraman (2024) demonstrated a detailed mechanism involving hyalgan-coated polymeric pullulan nanoparticles. The nanoparticles were characterized by zeta potential, Transmission Electron Microscopy (TEM), Scanning Electron Microscopy (SEM), and Fourier-transform infrared (FTIR) analyses. The coating of hyalgan on the nanoparticles not only contributed to the biocompatibility and stability of the formulation, but also influenced the particle size, encapsulation efficiency, and drug release kinetics. The presence of hyalgan improved drug release efficiency, reaching 95% within a specified timeframe, without inducing hemolysis or significant toxicity [96].

In summary, the specific mechanisms involving the molecular interactions of pullulan with drug agents, as exemplified in the mentioned studies, contribute to the improved characteristics of pullulan nanoparticles. These mechanisms include enhanced permeation and retention effects and specific interactions, such as hydrogen bonding interaction, hydrophobic interaction, and electrostatic interaction, which influence particle size, stability, encapsulation efficiency, and drug release kinetics. Such molecular insights underscore the significance of pullulan-based polymeric nanoparticles in advancing drug delivery systems with heightened precision and efficacy for biomedical applications. 

## 5. Challenges and Future Perspectives

### 5.1. Addressing Current Limitations and Challenges in Pullulan-Based Drug Delivery

The authors have identified several key observations based on the available literature on the development of pullulan-based nanoparticles for drug delivery. Each study employs a distinct approach, encompassing a range of fabrication techniques such as chemical synthesis and physical methods like nanoprecipitation, with unique combinations of polymers and active ingredients. While these methodologies offer specific advantages including scalability, control over particle size and morphology, and targeted drug delivery, they also present inherent limitations. For instance, chemical synthesis methods may involve the use of toxic reagents and require extensive purification steps, potentially impacting the biocompatibility of the final nanoparticles [96,97]. On the other hand, physical methods like nanoprecipitation may lack precise control over particle size distribution, leading to batch-to-batch variability [33,99]. Moreover, the choice of active ingredients, such as anticancer drugs or imaging agents, significantly influences the characteristics of the nanoparticles produced. Specific physicochemical properties of the drug such as poor aqueous solubility or instability within the nanoparticle matrix can also hinder their therapeutic effectiveness. 

Additionally, the characterization and in vitro evaluation of the formulated nanoparticles play a crucial role in assessing their suitability for drug delivery applications. Comprehensive insights into the physicochemical properties, drug release kinetics, and cytotoxicity profiles of the developed nanoparticle formulations are important [86,98]. If necessary, chemical modification and optimization may be conducted to further improve the physicochemical characteristics of the polymer, thus enhancing the cytotoxicity and overall drug release profile of pullulan-based nanoparticles [104,105]. The development of nanoparticle drug delivery systems utilizing pullulan polymer has been explored through diverse techniques.

### 5.2. Proposing Potential Solutions and Avenues for Future Research

In addressing the limitations described earlier, innovative solutions have been proposed by earlier researchers. For instance, to mitigate the challenges associated with chemical synthesis methods, alternative green synthesis routes could be explored. This includes employing eco-friendly reducing agents and capping agents, as demonstrated by Constantin et al. (2023), to enhance biocompatibility and reduce environmental impact [33]. Furthermore, to overcome the variability in the size of the nanoparticles produced through physical methods like nanoprecipitation, advanced techniques such as microfluidics could be employed. Microfluidic platforms offer superior control over reaction parameters, resulting in monodisperse nanoparticles with uniform size and shape, thus minimizing batch-to-batch variability. Additionally, to address the poor solubility or stability of active ingredients, novel strategies such as prodrug nanoparticle formulations have been proposed by Zhao et al. (2023) [98]. These formulations facilitate controlled drug release through stimulus-responsive mechanisms, ensuring enhanced therapeutic efficacy while minimizing adverse effects. Lastly, to improve the characterization and evaluation of nanoparticle formulations, the integration of advanced analytical techniques such as mass spectrometry imaging and single-particle tracking could provide valuable insights into drug release kinetics and cellular uptake dynamics, as demonstrated by Xu et al. (2022) and Tian et al. (2022) [86,104]. By integrating these innovative solutions, researchers can overcome the existing limitations and propel the development of more efficient and clinically viable pullulan-based nanoparticles for drug delivery.

## 6. Discussion and Author Perspective 

Nanoparticle drug delivery systems are an advanced method in modern medicine that aim to improve the effectiveness of drugs, minimize adverse effects, and enhance compliance among patients. These methods employ nanoparticles as carriers for delivering therapeutic drugs to precise target locations in the body, enabling controlled release and targeted delivery.

Pullulan, a polysaccharide obtained from fungi, has attracted interest because of its ability to interact well with living organisms, lack of toxicity, and capacity to break down naturally. Pullulan possesses features that make it very suitable for biomedical applications, especially in the context of nanoparticle drug delivery systems. Pullulan provides benefits such as the increased stability, controlled release, and greater solubility of encapsulated drugs.

Pullulan has undergone thorough research and development to be used as the main polymer matrix for nanoparticles. The incorporation of nanoparticles into medication formulations seeks to tackle multiple obstacles in drug delivery, such as improving solubility, modifying release patterns, enhancing therapeutic effectiveness, and ensuring stability. For example, nanoparticles made from pullulan have demonstrated potential in various fields such as cancer therapy and antimicrobial therapies. This is because they are capable of efficiently encapsulating and delivering drugs.

Several processes contribute to the efficiency of pullulan in nanoparticle drug delivery systems. The structure of pullulan enables the encapsulation of pharmaceuticals within its matrix, providing protection against degradation and enabling regulated release. In addition, pullulan-based nanoparticles utilize hydrogen bonding and electrostatic interactions to maintain nanoparticle stability and affect the release of drugs.

The characterization of pullulan-based nanoparticles includes the use of techniques such as Dynamic Scanning Calorimetry (DSC), X-ray Diffraction (XRD), Fourier-transform infrared spectroscopy (FTIR), as well as measurements of particle size and zeta potential. These approaches offer crucial knowledge about the physicochemical characteristics of nanoparticles, verifying their structure, crystallinity, chemical surface properties, and stability. For example, Differential Scanning Calorimetry (DSC) may detect thermal changes occurring in nanoparticles, X-ray Diffraction (XRD) can determine the crystalline structures present, and Fourier-transform infrared spectroscopy (FTIR) can confirm the chemical compositions and interactions.

Although pullulan-based nanoparticles offer benefits, their development encounters obstacles. These factors encompass variations in the distribution of particle sizes, challenges related to the ability to scale up production, and worries over the compatibility with living organisms in certain methods of synthesis. In order to address these restrictions, researchers are investigating different methods of creating something, fine-tuning the parameters used in the process, and improving the ways for analyzing the properties of the final product to ensure that it can be consistently reproduced and consistently perform well.

## 7. Conclusions

Overall, pullulan-based polymeric nanoparticles show great potential for enhancing drug delivery approaches. Through the utilization of its distinct characteristics and the optimization of formulation techniques, pullulan has the capacity to transform targeted drug delivery, providing improved therapeutic results while reducing negative consequences. Ongoing research and innovation in this field are essential for fully realizing the clinical promise of pullulan-based nanoparticle technology.

## Data Availability

Data will be made available on request.
